# Abdominal Wall Leiomyoma: A Case Report

**Published:** 2020

**Authors:** Leili Hafizi, Seyedeh Azam Pourhoseini

**Affiliations:** - Department of Obstetrics and Gynecology, School of Medicine, Mashhad University of Medical Sciences, Mashhad, Iran; - Imam Reza hospital, Mashhad University of Medical Sciences, Mashhad, Iran

**Keywords:** Abdominal wall, Leiomyoma, Mass, Smooth muscle tumor

## Abstract

**Background:**

Fibroma or leiomyoma is the most common benign tumor of the female reproductive system, which is usually found in the uterus, but may also occur in other places, such as the ovary, the broad ligament, and in rare cases in the abdominal wall. The formation of the abdominal wall leiomyoma may result from the implantation of myometrium tissue following surgical removal of the uterine leiomyoma, but sometimes these masses occur in a person who has no history of myomectomy.

**Case Presentation:**

This case was a patient who became a candidate for laparoscopy due to abnormal uterine bleeding and pain in the right upper quadrant of the abdomen and ovarian mass. The patient underwent laparotomy due to the inability of surgeons to insert the veress needle because of the presence of mass in the abdominal wall. The pathologic report of the abdominal mass was leiomyoma. This article has been approved by the Ethics Committee of the University (6562276).

**Conclusion:**

The formation of myoma on the abdominal wall is rare but given the fact that leiomyoma can be created at each part of the body with smooth muscles, including the anterior abdominal wall, this diagnosis should be considered for the differential diagnosis of abdominal masses.

## Introduction

Fibroma or leiomyoma is the most common benign tumor of the female reproductive system, with 20% of women suffering from the problem (1). The most common site for this tumor is uterus, but it may also occur in other places, such as the ovary, the broad ligament, and in rare cases in the abdominal wall (2).

Several theories have been presented to explain the cause of myoma formation in the abdominal wall. Generally, these myomas are divided into two primary and parasitic groups. Theories such as smooth muscle cell transformation and secondary metaplastic changes of non-muscle cells seem logical for the development of the primary myoma. In contrast, for parasitic myoma, the best explanation is the existence of a series of events that attach the uterine myomas to the anterior abdominal wall, and then the source of its blood supply is developed and its connection with the womb is separated (6).

Treatment of abdominal wall myoma is the complete removal of the mass and in case of full resection of the mass, the likelihood of recurrence is rare (11).

## Case Presentation

The case was a 25-year-old MG_3_L_1_ab_2_ woman with a history of infertility, complaining from irregular uterine bleeding and she was diagnosed with a pelvic mass in MRI. She got married 7 years ago.

All of her pregnancies were by induction and ovulation and the first and second pregnancies were aborted spontaneously between 6 and 8 weeks. The third pregnancy, 3 years ago, was terminated by cesarean section and resulted in the birth of a healthy baby.

**Figure 1.**
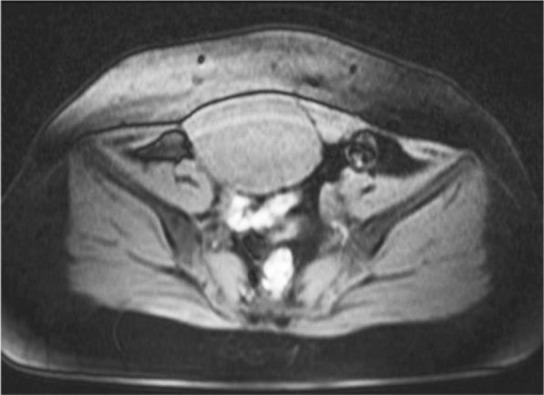
MRI view

The patient who had menorrhagia for the last 6 months was examined for irregular uterine bleeding. She also complained about occasional pains under the abdomen and in the right upper quadrant abdomen. In MRI, the retroperitoneal uterus and endometrial thickness was 5 mm, and a mass with an abnormal heterogeneous signal and heterogenic enhancement of about 95×80 *mm* in the anterolateral and right border of the hip with the extension to the hypogastric region was reported suggesting tumoral lesions in the right ovary or endometrium (Figure 1).

According to the report of the ovarian mass in MRI, the patient became a candidate for laparoscopy.

The laparoscopic surgery was planned in the operating room of Mehr Hospital in Mashhad on January 10, 2018. Initially, to enter the abdominal cavity, there was no possibility of passing the veress needle through abdominal wall from the umbilicus and the Palmer’s point, so the decision was made to enter the abdominal cavity through open laparoscopy. After creating a 2 *cm* incision in the umbilicus, and touching with finger, a solid and abnormal texture in this place was observed. Therefore, the decision was made to continue the operation by laparotomy.

**Figure 2.**
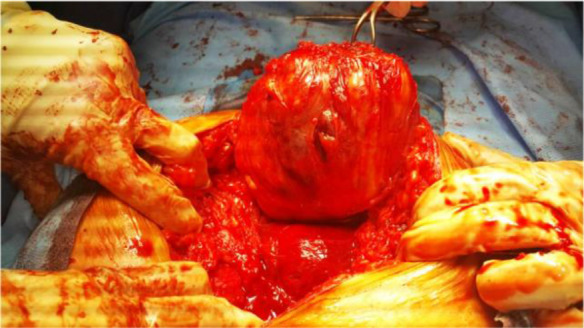
Abdominal wall leiomyoma

The abdomen was opened with a midline incision and a solid mass was in the midline of the abdominal wall adhering to the rectus muscles and the fascia with 12 *cm* in size. First, the mass was dissected from the rectus muscles and the peritoneum below it. After complete dissection, it was observed that the mass was connected to the abdominal wall with a relatively thick bundle (2 *cm*) in the suprapubic region (Figure 2).

The mass was similar to uterine fibroids in shape and consistency. After complete removal of the mass, in the examination of the abdominal wall, the visceral peritoneum was completely healthy. The visceral peritoneum was opened to check the abdominal cavity. No specific pathological findings were observed in the examination of the abdomen and pelvis. The uterus and adnexa were completely healthy and there was no evidence of adhesion, mass or ovarian cyst or uterine fibroids. So, the surgery was terminated.

Pathology report of the abdominal wall mass was leiomyoma.

## Discussion

Fibroma or leiomyoma is the most common benign tumor of the female reproductive system, with 20% of women suffering from the problem (1). The most common site for this tumor is the uterus, but it may also occur in other areas such as the ovary, the broad ligament, and in rare cases in the abdominal wall (2).

Uterine fibroids may be asymptomatic, or with symptoms such as abnormal uterine bleeding, pain, infertility, or signs of a compression of the myoma on adjacent organs (1). Myoma can originate from smooth muscles of the abdominal wall and, in rare cases, of smooth muscles of the stomach, intestine, artery wall, *etc*. The transformation of such cells into the myoma is due to somatic mutations and possibly some unknown hormonal synergistic functions, the disruption of lipid metabolism and local growth factors (3).

The most common primary diseases of the rectus muscle sheath are desmoid tumor and rectus sheath hematoma. Leiomyoma of the rectus muscle sheath is extremely rare (4).

The formation of myomas in the abdominal wall is rare, and is possibly due to myometrium tissues implantation after surgery for the removal of uterine leiomyoma, especially after laparoscopic myomectomy (5). In a case report from Moon et al., the incidence of abdominal wall myoma was reported three years after laparoscopic myomectomy (2).

There was no history of myomectomy in our patient, but the history of cesarean section was mentioned three years ago.

Several theories have been presented to explain the cause of myoma formation in the abdominal wall. Generally, these myomas are divided into two primary and parasitic groups. Theories such as smooth muscle cell transformation and secondary metaplastic changes of non-muscle cells seem logical for the development of the primary myomas. In contrast, for parasitic myoma, the best explanation is the existence of a series of events that attach the uterine myomas to the anterior abdominal wall, and then the source of its blood supply is developed and its connection with the womb is separated (6). The history of the reported patient did not indicate the presence of uterine myoma, so the parasitic myoma was discarded in this case, and it seems that the myoma of the abdominal wall developed lately in the patient.

The abdominal wall myoma shows the loss of fat between the rectus sheath and the lateral wall of the abdominal wall muscles, and is usually visible as a hypodense tissue without contrast enhancement (7). In our patient, heterogeneous mass with heterogenic enhancement was reported in the anterior and right border of the pelvis, while in a case report by Ono et al., in a CT scan performed for a patient with abdominal wall leiomyoma, the isotonic homogeneous mass was reported along with an enhancement near the anterior abdominal wall.

De novo leiomyoma of the abdominal wall refers to a condition in which the leiomyoma of the abdominal wall is created in a person who has no previous history of abdominal surgery. Theoretically, leiomyoma can be created anywhere in the body with a smooth muscle, including the anterior abdominal wall (8).

In our patient, there was a previous history of cesarean section, so she can not be placed in the group of de novo leiomyoma of the abdominal wall.

Somatic soft tissue myoma often appears with large local masses. Macroscopically, these myomas are manifested in the shape of masses with clear borders that are surrounded by a fiber false capsule (9). The presentation of the abdominal wall myoma in the patient was reported as abdominal pain and mass in the abdominal wall. In a case report by Gabriel et al., the manifestation of abdominal wall myoma in the patient was as a periumbilical mass and chronic abdominal pain (10).

Endometriosis lesions of the abdominal wall and desmoid tumors are more hyperdense than leiomyoma, and on the other hand, there is a lentiform shape on the abdominal wall myoma, while endometriosis lesions and desmoid tumors are round or oval masses (11). In the MRI of the patient, the differential diagnosis of the mass was reported as endometrium.

Performing an abdominal palpation or bimanual physical examination is one of the most important evaluations before surgery that can help the surgeon determine the location of the mass (12).

On the other hand, the surgeon’s ability to interpret radiological imaging, in case of incorrect report, leads to right decision to determine the surgical plan and prevent quick and incorrect decisions during surgery (12).

In a case report of Sel, a case of a 54-year-old (Parity: 4) female was reported. She was first admitted to the general surgery clinic with a complaint of vague abdominal pain. Abdominal ultrasound and computed tomography (CT) revealed the mass, measuring 10x9x7*cm*, located at the left adnexal area, reported as complex ovarian cyst. She had no history of an abdominal surgery. Laparoscopic surgery was planned. General observation of the abdominopelvic organs was done, but no ovarian cyst or pelvic mass was observed and uterus was normal. The surgeon looked for the mass which was revealed by ultrasound and CT. The surgeon located the mass with the help of abdominal palpation and laparoscopic observation at the same time, located anterior to the abdominal parietal peritoneum beneath the rectus muscle.

Pathology detected the mass as leiomyoma (12).

In our patient, failure to enter the abdomen for laparoscopy and laparotomy led to the discovery of the abdominal mass.

In radiology images, there was also a mass in the posterior surface of the rectus muscle.

Perhaps preoperative abdominal examination and accuracy in radiological images could help determine the proper surgical procedure and prevent confusion of the surgeon.

The treatment of the abdominal wall myoma is the complete removal of the mass, and in case of complete and successful resection, the probability of recurrence is rare (13). A complete resection of the mass was also performed for this patient and the mass was sent for pathology.

Meticulous examination of the mass’ parasitic blood supply is crucial to safe resection and good outcomes for patients (14).

## Conclusion

The formation of myoma is rare in the abdominal wall, but given the fact that the leiomyoma can be created at each part of the body that has smooth muscles, such as the anterior abdominal wall, it should be included in the differential diagnosis list of the abdominal wall masses. The treatment of the abdominal wall myoma is the complete removal of the mass, and in case of complete and successful resection, the probability of recurrence is rare.
